# The Annotation, Mapping, Expression and Network (AMEN) suite of tools for molecular systems biology

**DOI:** 10.1186/1471-2105-9-86

**Published:** 2008-02-06

**Authors:** Frédéric Chalmel, Michael Primig

**Affiliations:** 1 Institut National de la Santé et de la Recherche Médicale (Inserm) Unité 625, Groupe d'Etude de la Reproduction chez l'Homme et les Mammifères, Institut Fédératif de Recherche 140; Université de Rennes 1, Campus de Beaulieu, F-35042 Rennes, France

## Abstract

**Background:**

High-throughput genome biological experiments yield large and multifaceted datasets that require flexible and user-friendly analysis tools to facilitate their interpretation by life scientists. Many solutions currently exist, but they are often limited to specific steps in the complex process of data management and analysis and some require extensive informatics skills to be installed and run efficiently.

**Results:**

We developed the Annotation, Mapping, Expression and Network (AMEN) software as a stand-alone, unified suite of tools that enables biological and medical researchers with basic bioinformatics training to manage and explore genome annotation, chromosomal mapping, protein-protein interaction, expression profiling and proteomics data. The current version provides modules for (i) uploading and pre-processing data from microarray expression profiling experiments, (ii) detecting groups of significantly co-expressed genes, and (iii) searching for enrichment of functional annotations within those groups. Moreover, the user interface is designed to simultaneously visualize several types of data such as protein-protein interaction networks in conjunction with expression profiles and cellular co-localization patterns. We have successfully applied the program to interpret expression profiling data from budding yeast, rodents and human.

**Conclusion:**

AMEN is an innovative solution for molecular systems biological data analysis freely available under the GNU license. The program is available via a website at the Sourceforge portal which includes a user guide with concrete examples, links to external databases and helpful comments to implement additional functionalities. We emphasize that AMEN will continue to be developed and maintained by our laboratory because it has proven to be extremely useful for our genome biological research program.

## Background

High-throughput DNA sequencing, microarray-based mRNA expression profiling, proteomics experiments and protein-protein interaction assays have been yielding large and complex datasets that need to be integrated with functional information at the gene- or genome level. Large scale expression profiling using microarrays is among the most popular experimental approaches in genome biology and therefore optimized methods are available for all key analytical steps. They include raw data pre-processing, quality control and normalization [1,2], identification of differentially expressed genes during static or time-course conditions [3-5], gene clustering [6-8] and searching for significant over- or under-representation of functional annotation in expression clusters [9-11]. The *Bioconductor *project provides numerous software packages developed in *R *that are devoted to high-throughput analysis tasks [12-14]. However, installing and running them requires extensive programming skills that are not yet commonplace among life scientists. To alleviate this problem, programs with a convenient Graphical User Interface (GUI) have been developed that facilitate functional analyses in most cases limited to annotation by the Gene Ontology (GO) consortium [10] or restricted to a set of genomes [15,16]. Other tools correlate expression with chromosomal localization [17-21], protein-protein interaction [22] or pathway data [23].

In an attempt to combine analysis steps many web-based applications have been developed [24-30]. They are free and do not require maintenance work. However, their accessibility and speed depend upon web-traffic, server availability and the specifications of the analyses procedure. Moreover, web-based systems usually provide pre-configured and inflexible approaches to data analysis and often do not include advanced options to combine different types of high-throughput data. In order to address these issues and to allow for integrated exploration of different types of data we have developed the Annotation, Mapping, Expression, and Network (AMEN) program that enables users to explore and analyse multifaceted high-throughput biological data. It includes a suite of tools and algorithms for which parameters can be fine-tuned and analysis steps ordered as required. AMEN covers array data management, analysis and interpretation in a manner similar to EXPANDER 2.0 [29]. However, our software includes more options to combine different types of data and enables users to import, not only genome annotation and transcriptome-, but also proteome and interactome data without species restrictions.

## Implementation

**Figure 1:**
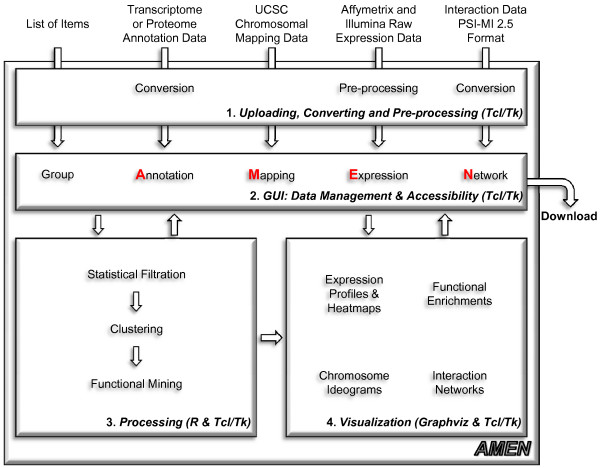
**The AMEN architecture**. A flow-chart diagram of the software and work-flow is shown.

**Figure 2:**
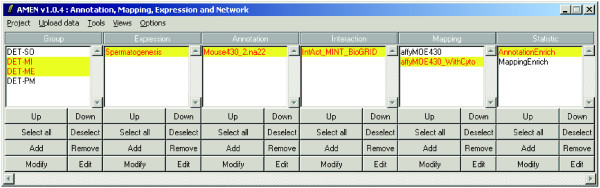
**The Main Application Window**. A screen shot of the main application window is given. A possible analysis strategy for mammalian testicular expression data is shown in the six data type panels as indicated. Four groups (clusters) of genes are defined as differentially expressed in testis and somatic (DET-SO), mitotic (DET-MI), meiotic (DET-MI) and post-meiotic (DET-PM) depending on peak expression in Sertoli cells, spermatogonia, spermatocytes and spermatids, respectively. The expression dataset (Spermatogenesis) was obtained with a GeneChip covering approximately 25000 protein-coding mouse genes for which an appropriate annotation file is selected (Mouse430_2.na22). To visualize the interaction network of proteins falling into two selected clusters (DET-MI and ME) information from three sources is combined (IntAct_MINT_BioGRID). To display the chromosomal localization of selected genes falling into given expression clusters files with gene coordinates are available with and without cytological bands (affyMOE430, affyMOE430_WithCyto). Users can choose from statistical analysis of GO term enrichment in clusters (AnnotationEnrich) or gene enrichment on chromosomes (MappingEnrich).

**Figure 3:**
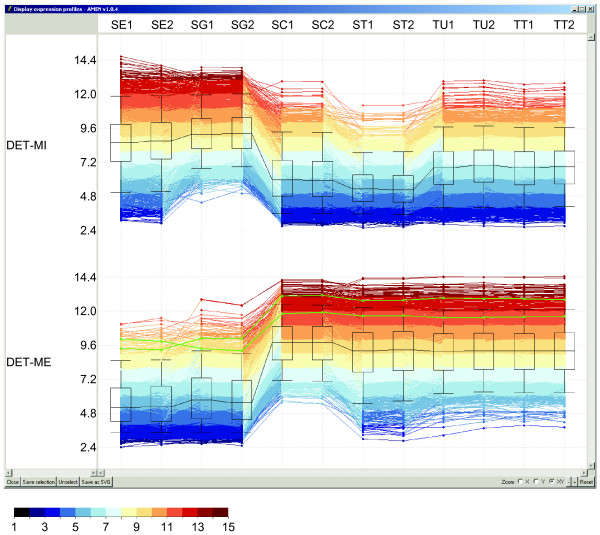
**Graphical display of expression profiling data**. Log2-transformed expression signal intensities are plotted against sample names on the Y- and X-axis, respectively. The signal distribution and the median are shown for each sample by a box plot. Data obtained for genes classified as differentially expressed in testis (DET) and showing peak transcription in mitotic (MI) or meiotic (ME) germ cells are displayed (see [33]). Sample names given in duplicate are Sertoli cells (SE), spermatogonia (SG), spermatocytes (SC), spermatids (ST), seminiferous tubules (TU), and total testis (TT). Lines and columns correspond to probe set ids and samples. Expression signals are shown in red (high) or blue (low) as indicated in the scale bar. Green lines represent expression profiles selected by the user.

**Figure 4:**
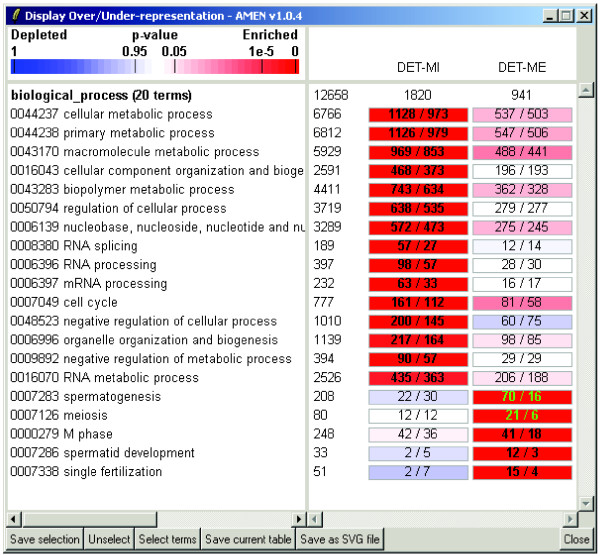
**Graphical output of GO term analysis**. An example of over-represented GO terms form the biological process category associated with genes from the DET-MI and -ME expression clusters is shown. The names of expression cluster and the numbers of genes are indicated on top of each column. The number of loci associated with a given GO term is shown to the left of the columns. Numbers of loci as observed and expected are given within color-coded rectangles with red and blue indicating over- and under-representation, respectively, according to the scale bar on top of the GO terms. Numbers in bold or green indicate significantly over-represented terms or genes selected by the user. To obtain the output shown we used an FDR-adjusted *p*-value of < 0.001, an OSIR > 0.1, and the minimum number of genes associated with one term was set to be > 10.

The AMEN software architecture consists of four layers implemented in Tcl/Tk (Figure 1) [31]. The first layer provides modules for uploading, formatting and pre-processing expression, annotation, chromosomal mapping and protein-protein interaction data. The second layer is the user-friendly GUI of the main application window that employs popup menus for *Project*, *Upload data*, *Tools*, *Views *and *Options *functionalities (Figure 2). Six panels provide access to lists of items such as probe IDs, genes, proteins (Group), and data from RNA/protein profiling experiments (Expression), genome annotation (Annotation), protein-protein and protein-DNA interaction (Interaction), chromosomal localization (Mapping) as well as the output of the statistical module (Statistic). Function buttons below each panel enable users to scroll item lists (Up/Down), mark items (Select all/Deselect), change the content of a panel (Add/Remove), change item names (Modify) or change the file content (Edit). Selected items in each panel highlighted in yellow are combined into different workflows by the user. For example, selecting mouse genes (Annotation panel) showing differential expression in testis (DET) and peak signals in somatic Sertoli cells (SO) compared to mitotic (MI), meiotic (ME) and post-meiotic (PM) germ cells (Group panel) and Spermatogenesis data (Expression panel) (see Figure 2) yields a graphical display of RNA profiling signals generated by the module controlled via the *Views > Expression data > Profiles *menu (Figure 3). Selecting protein network data (Interaction panel) enables users to display interaction patterns (see Figure 4 in reference [32]). Selecting chromosomal localization (Mapping panel) and statistical items enables users to correlate expression and mapping information or to reveal a link between transcriptional patterns and roles in biological processes (see reference [33] for more details). In the background, the third layer automatically creates and runs scripts for the statistical computing environment R which execute statistical calculations and clustering methods implemented in Bioconductor packages. The fourth layer displays the output based on Tk scripts and the graph rendering software GraphViz.

Our program requires Tcl/Tk, R and GraphViz programs available for all frequently used operating systems such as MS Windows, UNIX, Linux and Mac OS X to be pre-installed. Detailed downloading and installation instructions are accessible via the Sourceforge website. We also provide a Windows version of the software that pre-installs Tcl/Tk, R and GraphViz embedded into it. Note that AMEN supersedes goCluster, a much simpler tool previously developed in our laboratory [34]. We have decided to discontinue goCluster because it lacked key analysis features and the cost for further development and maintenance outweighed the benefit for our lab and the community. We emphasize that AMEN is frequently updated because this software is a key tool for our ongoing biomedical studies. Its structure greatly facilitates the implementation of new modules. Indeed, a single Tcl code line is sufficient to include additional functionalities into the main GUI.

## Results and Discussion

### Data uploading

The typical workflow involves five types of modules available in the current release: data uploading and pre-processing, statistical filtration, clustering, functional mining, and visualization. Data are imported and combined within an analysis project using the main application window (Figure 2). It includes six panels corresponding to different input data: items (such as genes, transcripts, proteins or probe identifiers), expression signal, functional annotation, (protein-protein) interaction, chromosomal location and statistical data. During this process items (such as probe set IDs) are automatically associated with other data types (including gene symbols, chromosomal position and GO terms). This interface makes it easy to access the data and to design an optimal analysis procedure. Data are input as text files in tab-delimited compatible format to ensure compatibility with all operating systems.

#### Group data

Users can upload pre-selected lists of items (called "main entries", such as probe, transcript, gene, or protein identifiers) or they can obtain such lists via statistical filtration, clustering, and/or visualization modules (see Figure 3 and 4).

#### Expression data

Currently, expression data quality control and normalization modules are implemented for commercial Affymetrix high density oligonucleotide microarrays (GeneChips) and Illumina Gene Expression BeadArrays. Methods for background correction, variance stabilization and normalization methods are MAS5.0, RMA, GCRMA and RSN [35]. It is also possible to upload pre-normalized expression datasets as long as they are represented as tab-delimited matrices whose rows and columns contain main items (usually probe identifiers) and experimental condition names, respectively.

#### Annotation, Interaction and Mapping data

Functional information for transcriptome (Affymetrix and Illumina CSV annotation files), proteome (International Protein Index, EBI and NCBI whole-proteome data files), interactome (Proteomics Standards Initiative-Molecular Interactions 2.5 files used by IntAct, MINT, and BioGRID) and chromosomal mapping analysis (PSL chromosomal location files from the UCSC web site) is imported and converted into the appropriate file format using a straightforward procedure [36-43].

### Statistical filtration

These modules output lists of significantly differentially expressed items (represented as transcripts or probe identifiers) identified within a given set of samples. Users can select transcripts showing strong variations across experimental conditions via threshold parameters including expression level cut-off, standard deviation or fold-change. Furthermore, a similarity search module helps retrieve groups of co-expressed transcripts using a specific user-defined pattern. Once a set of target transcripts is identified, the permutation (randomization), moderated t-test (empirical Bayes approach) and non-parametric rank-based statistic methods are employed to determine if changes in signal intensity are reproducible and significant. These methods are implemented in *multtest*, *samr, limma *and *RankProducts R *packages, respectively [44-46]. False positives are taken into account by adjusting p-values according to the Hommel (control of the family wise error rate) or Benjamini/HochBerg [determination of the False Discovery Rate (FDR)] multiple testing correction methods [47,48].

### Clustering

Clustering methods are used to classify items based on their overall degree of similarity across the experimental conditions. These algorithms are notably critical for the identification of genes that are co-expressed (showing similar patterns of transcription), co-regulated (sharing common promoter elements) or that play roles in a particular biological process. Users can choose between three hierarchical clustering modules: HCLUST (hierarchical), AGNES (AGglomerative NESting) and DIANA (DIvisive ANAlysis) [8,49]. Four supervised partitioning methods include k-means [50], PAM (Partitioning Around Medoids), FANNY (Fuzzy Analysis Clustering) and CLARA (Clustering LARge Applications) [49]. We also included two unsupervised clustering modules called MCLUST (Model-based CLUstering) and HOPACH (Hierarchical Ordered Partitioning and Collapsing Hybrid) that automatically determine the number of clusters in a given dataset [51,52]. Finally, to estimate the quality of the classification or to help identify the optimal number of clusters that yields the best separation of different expression patterns the silhouette plot method is available [53].

### Functional mining

Expression clusters are validated and further analysed by searching for over- or under-represented functional annotation terms associated with the items (genes) in these clusters using hypergeometric or binomial statistical tests. *p*-values are adjusted using multiple testing correction methods as described above. Functional information is most often provided by the GO consortium [9] but, in principle, AMEN can process data from any source of information present in the uploaded annotation files such as InterPro protein domains, biochemical pathways, chromosomal mapping data or other information provided by the user.

Note that ontology vocabularies have a hierarchical structure such that an item (e.g. gene) is associated with multiple redundant terms. To reduce the annotation term output we employ the Ontology Specific Information Rate (OSIR): *OSIR *= (*n*-*m*)/*n*, where *n *and *m *are the numbers of items associated with a given over-represented term (parent node) and associated to its subordinate over-represented terms (child nodes) respectively. The minimal *OSIR *threshold value is typically set between 0.05 and 0.20 which means that if less than 5% or 20% of the genes associated with a given parent node are not related with its child nodes, the parent node is eliminated.

### Data visualization

**Figure 5:**
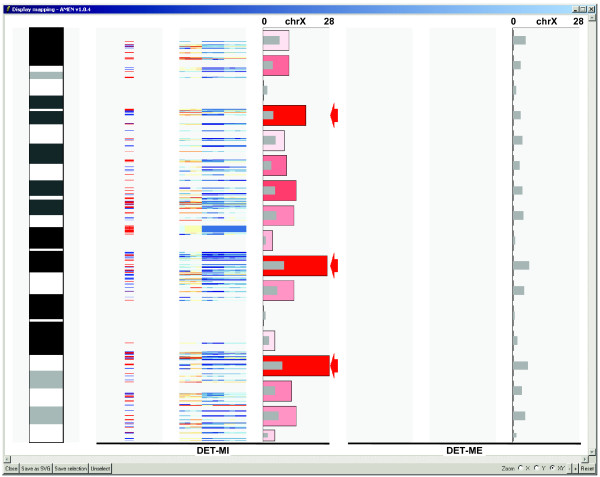
**Chromosomal ideogram representation**. An ISCN ideogram of the mouse X chromosome is shown (column 1). The chromosomal localization of genes in the DET-MI expression cluster is marked by red (plus or top DNA strand) and blue (minus or bottom DNA strand) lines (column 2). A color coded heat map (see scale bar in Figure 3) shows expression signals for each sample (column 3). The numbers of mapped genes within consecutive regions of 10 Mbp are plotted on the X- and Y-axes, respectively (column 4). Color coded bars show the numbers of observed loci with red and blue indicating over- or under-representation. Grey bars represent the number of loci falling into a given region by chance. Red arrows mark regions that are enriched in loci (FDR-adjusted *p*-value < 0.001). The remaining columns 5–7 show that the X-chromosome is devoid of meiotic genes falling into the DET-ME cluster.

**Figure 6:**
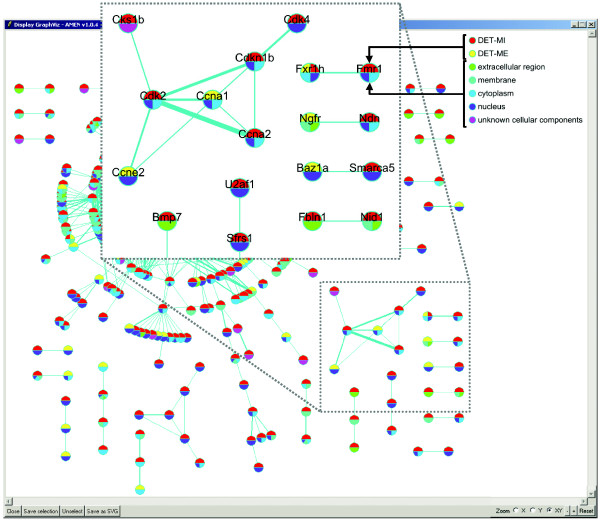
**Display of Protein-protein interaction networks**. A global view of protein-protein interactions based on combined mouse, rat and human data retrieved from IntAct, MINT and BioGRID databases is shown. Blue lines connecting nodes (proteins) represent direct physical interactions. Line thickness increases with the number of published observations supporting the predicted interaction. Nodes are color coded to indicate the expression cluster the protein belongs to (top half) and the sub-cellular component to which it localizes (bottom half) as shown.

Four types of visualization modules are currently implemented. First, users can display expression data as false color-coded heat maps or as graphs (Figure 3). Second, a color-coded graphical module to display significantly over- or under-represented functional annotation terms among clusters (Figure 4). It is possible for this output to contain data from multiple experiments and simultaneously display as distinct columns. Alternatively, it is possible to display over-represented *GO *terms and their related parent nodes as directed acyclical graphs. Third, a module is included to create chromosome ideograms according to the International Standard on Cytogenetic Nomenclature (ISCN) (Figure 5). This functionality helps reveal correlations between expression patterns and chromosomal mapping of selected target items. It includes ISCN ideograms together with heat maps of expression data and histograms showing observed and expected numbers of genes in a given region. Finally, the complete set of *GraphViz *tools to draw network graphs of protein-protein or other types of interactions is available (Figure 6). Nodes representing biological items (proteins, genes) are color-coded to facilitate the interpretation of relationships between expression clusters, sub-cellular location and interaction data.

The visualization modules output interactive and clickable images providing detailed information for each cluster and gene that can be manually selected for further analysis. They also provide manual zooming (in the X, Y or both directions) and panning features enabling users to focus on specific results of interest. Finally, users can employ the Scalable Vector Graphics format (SVG) for viewing with all web browsers and further processing with SVG editors such as Inkscape or Adobe Illustrator [54,55].

### Data export

AMEN provides a module to export selected lists of items as an HTML table file which can be opened and further processed in spreadsheet applications such as MS Excel. Users can select different types of annotation, mapping and expression data and determine their order within the file to be exported.

### Application of AMEN

The program was critical for our study of the testicular expression program in human and rodents where a clear correlation between germline expression and reproductive function was established [33]. Note that this study included work on the negative correlation between meiotic gene expression and X-chromosome localization (see Additional file 1). Furthermore, we have used AMEN to compare testicular transcriptome and proteome data and to explore the protein-protein interaction network of gene products differentially expressed between testicular somatic and germ cells [32].

Ongoing work includes the expression signature of high infertility risk associated with undescended testes (Hadziselimovic et al., in revision), high-throughput analysis of mRNAs and proteins present in residual bodies (Rolland et al. and Brun et al., unpublished) as well as enrichment of functional annotation among the target genes of Abf1, an essential budding yeast DNA binding transcription factor (U. Schlecht and M. Primig, in press), and Ume6, a regulator involved in mitotic repression of meiotic genes in *S. cerevisiae *(T. Walther and M. Primig, in preparation). Our software is thus suitable for molecular systems biological data analysis combining data on DNA, mRNA and proteins across different species.

### Comparison to other solutions

Since AMEN is a freely available standalone molecular systems biology analysis tool we have compared it to typical examples of such software and not to web server-based applications that, in our opinion, are often less flexible and less complete than locally installed programs. Most available local solutions are R packages such as affylmGUI and illuminaGUI which provide quality control (QC), pre-processing, statistical tests and clustering applicable to Affymetrix GeneChip and Illumina BeadArray data, respectively [56,57]. BRB-ArrayTools is an MS Excel plug-in providing advanced statistical tests for the identification of differentially expressed genes, GO term enrichment and the option to expand functionalities using external R-scripts [58]. AMDA provides various QC, normalization, statistical and clustering functionalities and also includes a GUI, as well as GO term and KEGG enrichment [59]. As compared to these solutions our software has useful additional features such as an elaborate main application window facilitating work-flow management, sophisticated graphical output of (for example) GO term enrichment, cross-microarray platform compatibility, proteomics data import functionality, protein-protein and protein-DNA network data processing, and chromosomal localization and enrichment (Additional file 1). Finally, the graphical output of AMEN is interactive and enables users to sub-select and save lists of items.

### Future development

AMEN is regularly updated with new functionalities and modules. We intend to include, in the near future, data management for molecular pathway databases such as the Kyoto Encyclopaedia of Genes and Genomes (KEGG) in order to display metabolic pathways combined with protein-protein interaction and expression data [60]. We also plan to implement additional data pre-processing and normalization algorithms for the most recent generations of all-exon [61] and tiling microarrays [62,63] as well as a novel Principle Component Analysis (PCA) statistical module. Finally, we will integrate AMEN with MIMAS [64], our own solution for array data management and annotation to provide our laboratory and the community with a complete package for storing, describing, analysing and interpreting high-throughput data.

## Conclusion

AMEN facilitates the design and execution of optimized procedures for processing, analysis and interpretation of multifaceted high-throughput data. Key advantages include: an intuitive GUI, flexible design of transcriptome and proteome analyses strategies; and convenient interactive graphical output of results on expression signals, chromosomal mapping, functional annotation and network interactions. The modular structure allows for easy extension and customization. We will continue development and support of AMEN as an integral part of our long-term biomedical research program. The source-code is freely available for members of the bioinformatics community who wish to add their own functionalities.

## Availability and requirements

• **Project name: **AMEN

• **Project home page: **

• **Operating system(s): **Platform independent

• **Programming language: **Tcl/Tk, R, GraphViz

• **Other requirements: **ActiveTcl version 8.4.16.0, R version 2.6.0, GraphViz version 2.14.1 or higher

• **License: **GNU GPL

## Authors' contributions

FC initiated, developed the software and drafted the manuscript. MP contributed to the concept and wrote the manuscript. All authors read and approved the final manuscript.

## Supplementary Material

Additional file 1 Comparison of AMEN and other solutions. Comparison of features implemented in AMEN and other standalone solutions for high-throughput data analysis and interpretation. Corresponding references are given in the main text. An asterisk indicates that the program includes a given feature while a minus is put when the functionality is lacking. Click here for file
