# Supplementary material for: The Annotation, Mapping, Expression and Network (AMEN) suite of tools for molecular systems biology
Source: BMC Bioinformatics. 2008 Feb 6;9:86. doi: 10.1186/1471-2105-9-86 (PMC2375118; doi:10.1186/1471-2105-9-86)
Supplement: Additional file 1 — Comparison of AMEN and other solutions. Comparison of features implemented in AMEN and other standalone solutions for high-throughput data analysis and interpretation. Corresponding references are given in the main text. An asterisk indicates that the program includes a given feature while a minus is put when the functionality is lacking. [file 1471-2105-9-86-S1.doc]

**Table 1: **Comparison of features implemented in AMEN and other standalone solutions for high-throughput data analysis and interpretation. Corresponding references are given in the main text. An asterisk indicates that the program includes a given feature while a minus is put when the functionality is lacking.****

|  | AMEN | affylmGUI | illuminaGUI | BRB-ArrayTools | AMDA |
| --- | --- | --- | --- | --- | --- |
| QC | * | * | * | * | * |
| Pre-processing | * | * | * | * | * |
| Statistical tests | * | * | * | * | * |
| Clustering | * | * | * | * | * |
| GO term enrichment | * | - | - | * | * |
| User-friendly GUI | * | - | - | - | * |
| Chromosomal enrichment | * | - | - | - | - |
| Chromosomal localization | * | - | - | - | - |
| Multi-array compatibility | * | - | - | - | - |
| Proteomics data | * | - | - | - | - |
| Protein network data | * | - | - | - | - |
| Interactive graphics | * | - | - | - | - |
